# Maintaining stability of the rumen ecosystem is associated with changes of microbial composition and epithelial TLR signaling

**DOI:** 10.1002/mbo3.436

**Published:** 2017-01-21

**Authors:** Hong Shen, Zhan Chen, Zanming Shen, Zhongyan Lu

**Affiliations:** ^1^ College of Life ScienceNanjing Agricultural UniversityNanjingJiangsuChina; ^2^ Bioinformatics CenterNanjing Agricultural UniversityNanjingJiangsuChina; ^3^ Key Lab of Animal Physiology and BiochemistryCollege of Veterinary MedicineNanjing Agricultural UniversityNanjingJiangsuChina

**Keywords:** butyrate infusion, microbe–host interaction, rumen ecosystem, rumen epithelium, toll‐like receptors

## Abstract

We used the goat as a model to study the effects of rumen microbial composition and epithelial TLR signaling on maintaining rumen stability during exogenous butyrate interference. Six cannulated goats received a rapid intraruminal infusion of 0.1 mol/L potassium phosphate buffer with (BT,* n* = 3) or without (CO,* n* = 3) 0.3 g/kg·BW·day sodium butyrate for 28 days. The ruminal pH and the concentration of total SCFA were not affected by the interference. 16S rRNA gene amplicon sequencing revealed a change in microbial composition after the butyrate infusion. LEfSe analysis showed a shift of the biomarker species from butyrate‐producing bacteria to acetate‐and propionate‐producing bacteria. Quantitative PCR‐based comparisons showed that significant increases in TLR2, TLR5, and MyD88 expression were accompanied by a significant decrease in IL‐1β and IFN‐γ expression in the ruminal epithelium. Constrained correlation analysis showed that the relative abundance of *Roseburia* was positively correlated with the expression of TLR5. Taken together, our study shows that microbial composition plays an important role in maintaining the stability of the microbial ecosystem in rumen, and indicates that the microbe‐TLR‐cytokine axis was involved in maintaining the stability of the gastrointestinal ecosystem.

## 1 INTRODUCTION

Stability refers to the ability of an ecosystem to maintain the homeostasis of its ecological environment and function after interference (Pfisterer & Schmid, 2002). Stability is crucial for maintaining the metabolic processes of the resident microbiota under different conditions (Loreau et al., 2001). Loss of stability may lead to an impairment of the ecological function, and, moreover, a collapse of the system.

Thus far, it is unknown how the stability of the gastrointestinal (GI) ecosystem can be maintained. Experimental studies of soil microbial ecosystems have indicated that microbial composition is an important factor in maintaining the stability of microbial ecosystems. The functional characteristics of the individual species within a community play significant roles in accomplishing the functions of the ecosystem (Cragg & Bardgett, 2001). The epithelium is another important component of the GI microbial ecosystem. The microbiota constantly communicates with the epithelial immune system. Studies of monogastric animals have shown that the interactions between specific microbes and toll‐like receptors (TLRs) play important roles in shaping the composition of the GI microbiota (Jacobs & Braun, 2014). Using recognition of microbes with special microbe‐associated molecular patterns (MAMPs), TLR signaling suppresses the production of proinflammatory cytokines, leading to the promotion of the residence of nonpathogenic microbes in the GI ecosystem. In the intestinal tracts of mice, the deletion of MyD88, a critical component of the TLR signaling pathway, was associated with a reduction in segmented filamentous bacteria (SFB) (Larsson et al., 2012). We speculated that some unique microbes and their interactions with TLRs play important roles in the maintenance of the stability of the GI ecosystem.

The rumen is an organ located between the esophagus and the third stomach of ruminants. It is an ideal laboratory for elucidating the fundamental principles of the GI ecosystem because the physiological functions of the rumen epithelium and the composition of the rumen microbiota are similar to those of the human colon (Gressley, Hall, & Armentano, 2011). Additionally, it is easier to modulate the rumen microbiota than the colonic microbiota using a feeding strategy. For any experiment using rumen microbiota, the administration of the interference and the administration frequency and period can be accurately controlled. Furthermore, the study of Liu, Bian, Zhu, and Mao (2015) suggested the existence of a microbe‐TLR‐cytokine axis in the rumen. Therefore, we used goat rumen as a model to investigate the responses of the rumen microbiota to a long‐term butyrate infusion, and we investigated the correlation between the changes of the microbial composition and the expression of TLRs at the apical surface of the rumen epithelium.

This study allowed us to understand how the stability of the ruminal ecosystem is maintained and whether the microbe‐TLR‐cytokine axis is involved in this process.

## 2 EXPERIMENTAL PROCEDURES

### 2.1 Ethics

All management and experimental procedures were conducted according to the Guidelines for the Care and Use of Animals of Nanjing Agricultural University, 1999.

### 2.2 Animals

Six male goats (Boer × Yangtze River Delta White, 4‐month‐old) fitted with ruminal cannulas were randomly assigned to two groups: a control group (CO, *n* = 3) and a butyrate infusion group (BT, *n* = 3). Two hundred grams of concentrate was provided to the goats of both groups in two equal amounts at 0800 and 1700 daily. Hay and water were provided ad libitum. The chemical compositions of the dietary components are presented in Table S2. All goats received one dose intraruminally of 0.1 mol/L potassium phosphate buffer (50 ml) without (CO) or with (BT) approximately 0.3 g/kg body weight sodium butyrate (Merck, Hohenbrunn, Germany) at 0700 daily, that is, 1 hr before the morning feeding. After infusion, the rumen content was thoroughly mixed to ensure the uniform distribution of the infusions throughout the rumen. The experiment lasted for 28 days.

### 2.3 Sample collection

The ruminal fluid was taken on day 28 at 0 hr, 1 hr, 2.5 hr, 5 hr, and 8 hr after the butyrate infusion. An aliquot (20 mL) of ruminal fluid was strained through a 4‐layer cheesecloth and immediately subjected to pH measurement. Thereafter, a 5% HgCl2 solution (1 ml) was added, and the sample was stored at −20°C for the determination of the SCFA concentration. All goats were slaughtered 8 hr after the butyrate infusion on day 28. Immediately after slaughter, approximately 5 ml ruminal fluid was collected for the microbiota analysis. Rumen tissue from the ventral blind sac was quickly excised and washed repeatedly using ice‐cold PBS (pH 7.4) until the PBS was clear. The epithelium was separated from the muscle layers and stored at −80°C until RNA extraction. The ruminal SCFA concentration was determined using a chromatograph (HP6890N, Agilent Technologies, Wilmington, DE) as described by Yang, Shen, and Martens (2012).

### 2.4 Quantitative PCR

The total RNA was extracted from the ruminal epithelium using the RNeasy Mini Kit (Qiagen, Shanghai, China). A random hexamer primer (Invitrogen, Shanghai, China) and M‐MLV (Moloney murine leukemia virus) reverse transcriptase (Fermentas, Burlington, ON, Canada) were used to synthesize the cDNA. Quantitative PCR was performed using the StepOne Plus real‐time PCR system and software (Applied Biosystems, Den Ijssel, The Netherlands) and SYBR‐Green (Applied Biosystems) for detection. GADPH was chosen as the housekeeping gene. The primers of the targeted genes were designed according to the available sequences in NCBI (Table S3). The amplification efficiency of the primers was determined using a dilution series of epithelial cDNA. All samples were run in triplicate, and the data were analyzed according to the 2^−ΔΔCT^ method. The identity and purity of the amplified product were checked using analysis of the melting curve carried out at the end of the amplification (Livak & Schmittgen, 2001).

### 2.5 Ruminal microbiota analysis

The metagenomic DNA of the microbiota was extracted from the ruminal fluid using a Bacterial DNA Kit (Omega). The DNA concentration was determined using a Nanodrop 1,000 and stored at −20°C until further processing. The amplicon library preparation was performed using PCR amplification of the V3–V4 region of the 16S rRNA gene using the universal primers 338F (5′‐ACTCCTACGGGAGGCAGCAG‐3′) and 806R (5′‐GGACTACHVGGGTWTCTAAT‐3′) (Mori et al., 2014), including TruSeq adapter sequences and indices and AccuPrime Taq high fidelity DNA Polymerase (Life Technologies, Carlsbad, CA). All libraries were sequenced using an Illumina MiSeq platform (Illumina, San Diego, CA) at Biomarker Technologies, Beijing, China.

Paired reads were filtered for quality (Q30) and joined using FLASH version 1.2.11 (Magoč et al., 2011). Sequences that contained read lengths shorter than 400 bp were removed and classified into taxa by blasting using the Ribosomal Database Project (RDP) Database at a 97% similarity threshold. OTUs were hierarchically summed at all taxonomic levels, and the counts were normalized to relative abundance for each sample. The diversity of the microbial communities was estimated using the R program phyloseq package (McMurdie & Holmes, 2013). For a deeper analysis of the diversity of the major evolutional clades in the ruminal microbiota, all data were filtered to require a relative abundance of at least 1% in at least one sample. Then, MUSCLE version 3.8.31 (Edgar, 2004) was used to align the complete 16S rRNA sequences of the corresponding species in the RDP database, and RAxML version 8 (Stamatakis, 2014) was used to construct the phylogenetic tree. The R program ape package Paradis, Claude, & Strimmer, 2004 was used to plot the tree.

To identify significantly different OTUs between the groups, a linear discriminant analysis (LDA) with LEfSe (Segata et al., 2011) was used. The relationships between the abundance of each biomarker genus and the expression of the host genes were explored using the canonical correspondence analysis (CCA) of the vegan package (Oksanen et al., 2016). The genera used in the CCA analysis were significantly different by *t* test (*p *<* *.05). The R program ggplot2 package (Wickham, 2009) was used to generate the visual interpretation (biplot) of the gene–microbiota relationships. The coordinates of the arrows on the plot were determined using the expression of the genes, and the coordinates of the points were determined using the frequency of the genera.

## 3 RESULTS

### 3.1 Microbial metabolisms

**Figure 1:**
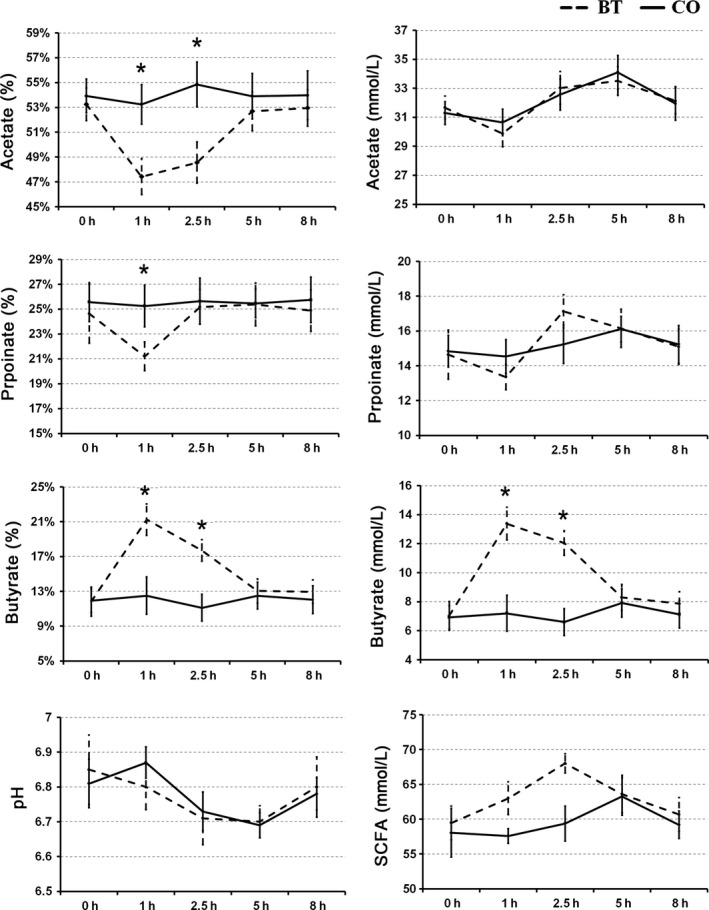
Effect of ruminal butyrate infusion on the concentrations of short‐chain fatty acids (SCFA; acetate, propionate, and butyrate), their molar proportions, total SCFA concentration, and the pH in the ruminal fluid of goats. 0 indicated the sampling time just before butyrate infusion, and other numbers indicated the sampling time after the butyrate infusion. “*” indicated *p* <.05 in *t* test

Before matutinal butyrate infusion, the concentration of the short‐chain fatty acid (SCFA) did not differ between the BT and CO groups (*p *>* *.05) (Figure 1). Additionally, there was no significant difference of total SCFA between the groups at investigated time points (*p *>* *.05). In the BT group, the molar proportions of butyrate increased (*p *<* *.05) by approximately 100% at 1 hr after the infusion compared with the preinfusion level (baseline). The molar proportions of butyrate returned to baseline at 5 hr. The molar proportions of acetate and propionate of the BT group were lower (*p *<* *.05) at 1 hr and 2.5 hr after infusion; then, the molar proportions gradually returned to their baseline levels. In the CO group, the molar proportion of the individual SCFAs did not significantly differ (*p *>* *.05) across time. There was no significant change in rumen pH between the groups (*p *>* *.05) across time.

### 3.2 Microbial composition

**Figure 2:**
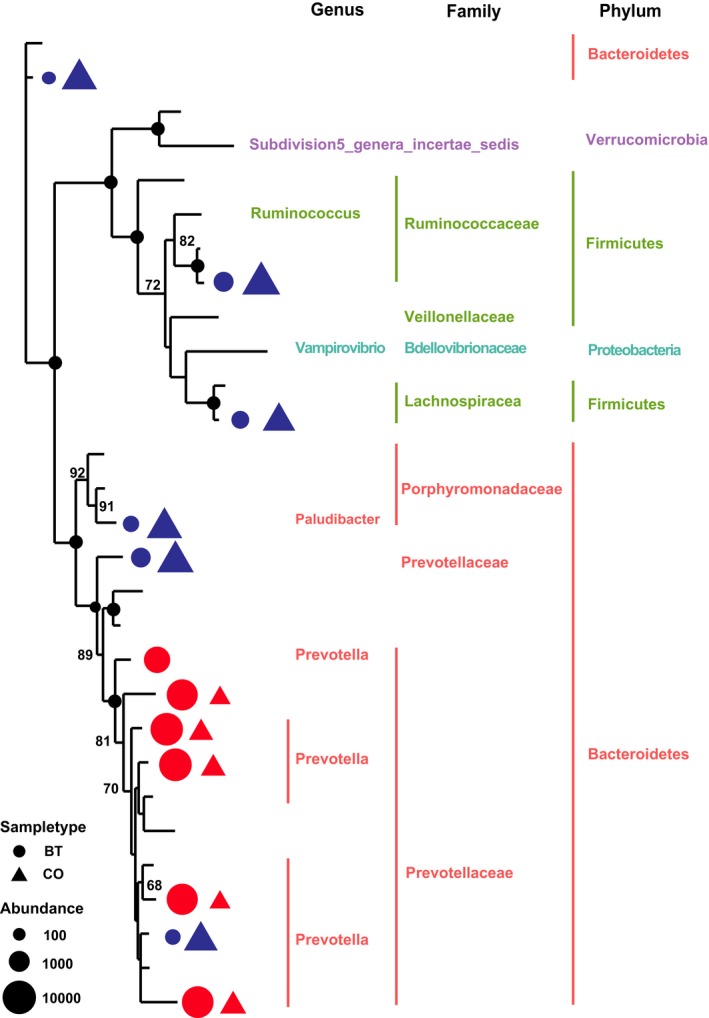
Maximum likelihood tree of the detectable OTUs (relative abundance > 1% for a given sample). The complete 16S rRNA gene sequence of the corresponding species in RDP database were used to construct the tree. Triangle indicates the OTUs in CO group, and the circle indicates the OTUs in BT group. Only the OTUs with significant differences (*p *<* *.05) in relative abundance are shown behind the branch. The size of the symbol indicates the relative abundance of OTUs. The red indicates a significant expansion (*p *<* *.05) on the relative abundance of the OTU after butyrate infusion, and the blue indicates a significant reduction (*p *<* *.05) on the relative abundance of the OTU after butyrate infusion. The bootstrap values, which are more than 60 in the ML analysis, are shown on the tree. The solid black circles on the nodes stand for the bootstrap value of 100

At the phylum level, a total of 18 prokaryotic phyla were identified using the RDP classification at a 97% similarity threshold, and 13 phyla were common to both groups. Bacteroidetes (63.9%–79.4%), Firmicutes (11.2%–26.6%), and Verrucomicrobia (3.3%–4.2%) were the most abundant of all bacterial phyla (Table 1). Chlorobi, Chloroflexi, Armatimonadetes and Gemmatimonadetes were only detected in the CO group, whereas Elusimicrobia was only detected in the BT group. At the genus level, a total of 117 genera were detected in the sequences, and 58 genera were common to all groups. The relative abundances of all the genera of the two groups are shown in Table S1. *Prevotella* was the most affected of all genera, as its abundance increased by 132% after the butyrate infusion. Moreover, the abundance of *Paraprevotella* increased by 83% after the butyrate infusion (Table 2). However, one OTU belonging to *Prevotella* exhibited discordant shift of its relative abundance (Figure 2). The nonmetric multidimensional scaling (NMDS) plot and the analysis of similarities (ANOSIM) revealed a divergence of the community structure of the two groups and also demonstrated the evident impacts of the butyrate infusion (Figure S1).

**Table 1:** Comparisons of the relative abundance of prokaryotic phyla in group BT and CO

Phylum	Group BT	Group CO	BT/CO
Bacteroidetes	79.4%	63.9%	1.24
Firmicutes	11.2%	26.6%	0.42
Verrucomicrobia	3.3%	4.2%	0.79
Proteobacteria	1.6%	1.7%	0.94
Lentisphaerae	1.4%	1.4%	1.00
Others	3.2%	2.3%	1.39

**Table 2:** Comparisons of the relative abundance of prokaryotic genera in groups BT and CO

Genus	Group BT	Group CO	BT/CO
*Prevotella*	27.4%	11.8%	2.32
Subdivision five genera incertae sedis	3.3%	4.2%	0.79
*Paraprevotella*	1.1%	0.6%	1.83
*Vampirovibrio*	1.1%	1.3%	0.85
*Lachnospiraceae*	1.0%	1.5%	0.67
*Ruminococcus*	0.4%	1.3%	0.31
*Barnesiella*	0.2%	0.8%	0.25
*Paludibacter*	0.2%	3.0%	0.07
Others	65.3%	75.5%	0.86

### 3.3 Diversity and abundance of the microbiota

As indicated by the Shannon and Simpson indices, the diversity of the community was not significantly different between the groups (Figure S1). The phylogenetic analysis of 29 detectable OTUs (relative abundance >1%) revealed two major clusters. The larger cluster consisted of the OTUs belonging to Bacteroidetes. The other cluster consisted of the OTUs belonging to Verrucomicrobia, Firmicutes, and Proteobacteria. In comparing the relative abundance of the OTUs between groups, a significant reduction in relative abundance was observed for Ruminococcaceae and Lachnospiraceae within the polyphyletic Firmicutes, and in Porphyromonadaceae and Prevotellaceae within the paraphyletic Bacteroidetes. Conversely, a significant expansion of the relative abundance was observed in Prevotellaceae within the larger cluster (Figure 2). However, all of these OTUs unexceptionally appeared in both groups, indicating a similar variety of the major OTUs between the groups.

### 3.4 Biomarker genera within the microbial community

**Figure 3:**
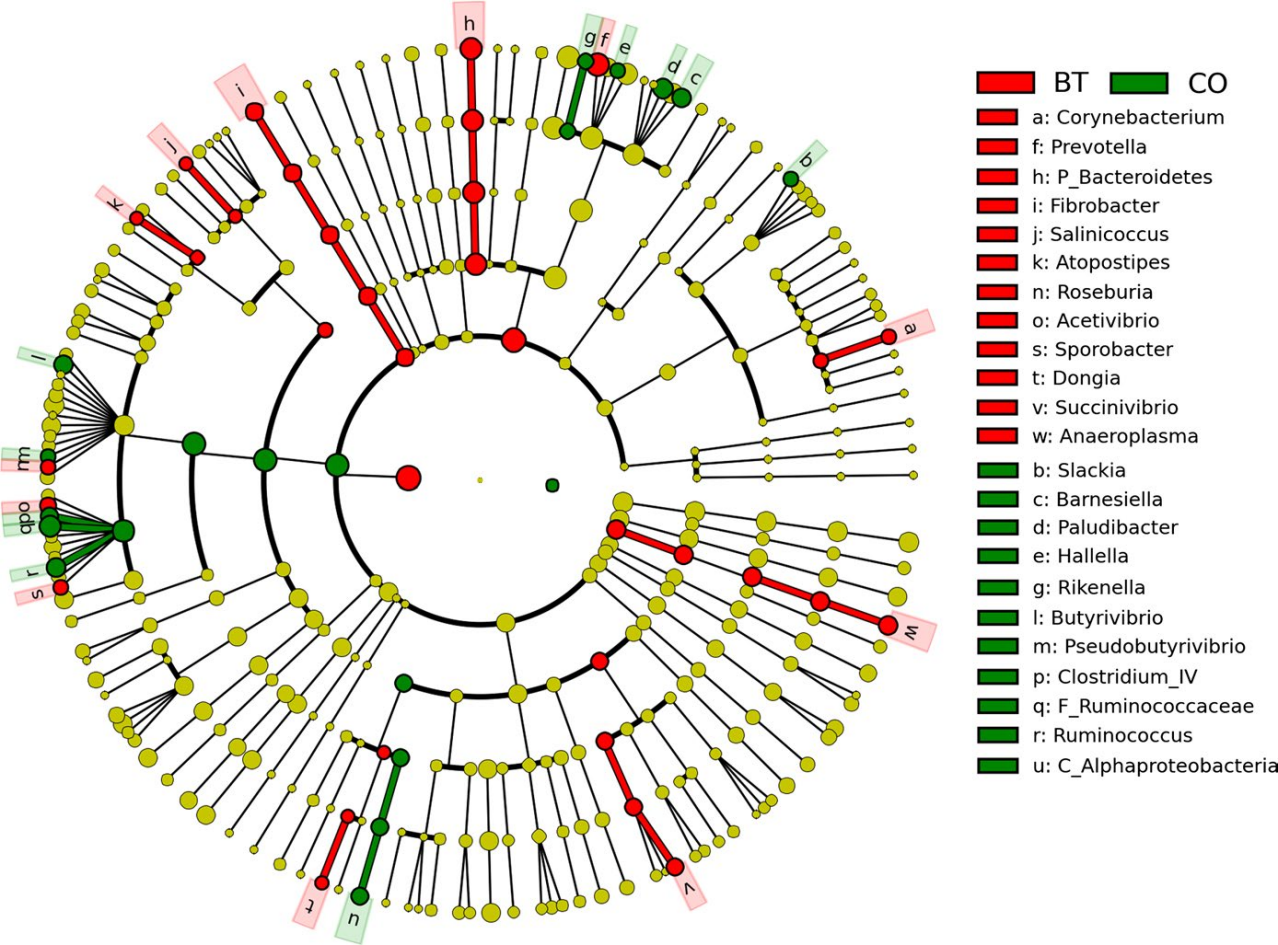
LEfSe analysis indicated the biomarker genera of the microbial community in different groups

LEfSe combined rank sum tests and taxonomic information to find the biomarker species with the greatest impact on the structure of the community. In our study, 12 genera were selected as biomarkers for the BT group, and 11 genera were selected as biomarkers for the CO group. The list of the biomarker genera is shown in Figure 3.

### 3.5 mRNA expression of the genes of the microbe‐TLR‐cytokine axis

Quantitative PCR‐based comparisons of the epithelial TLRs and cytokines showed that, compared with the CO group, the mRNA expression of TLR2, TLR5, and MyD88 of the BT group increased significantly (*p *<* *.05). Conversely, the expression of IL‐1β and IFN‐γ were significantly decreased (*p *<* *.05). The expression of TLR1, TLR4, TLR6, TLR10, IL‐6, and TNF‐α did not change significantly between the groups (Table 3).

**Table 3:** Comparisons of the expressions of genes located on the TLR‐cytokine axis using 2^−ΔΔCT^ method. All analyses were performed in triplicate. MSE: mean squared error

Genes	Group BT	Group CO	MSE	*p* value
TLR1	0.95	1.01	0.07	.733
TLR2	1.62	1.00	0.04	.009
TLR4	0.92	1.01	0.06	.055
TLR5	1.55	1.00	0.04	.025
TLR6	0.95	1.00	0.04	.538
TLR10	1.02	1.00	0.04	.819
MyD88	1.49	1.01	0.08	.011
IL‐1ß	0.79	1.01	0.04	.015
IL‐6	1.10	1.00	0.04	0.167
IL‐10	1.07	1.01	0.06	.527
TNF‐α	0.95	1.01	0.07	.176
IFN‐γ	0.60	1.01	0.05	.039

GAPDH (glyceraldehyde 3 phosphate dehydrogenase) was used as the housekeeping gene.

TLR = toll‐like receptor; MyD88 =  myeloid differentiation primary response 88; IFN‐γ  =  interferon‐gamma; IL = interleukin; TNF‐α  =  tumor necrosis factor alpha.

### 3.6 Correlation between the expression of TLRs and the abundance of biomarkers

**Figure 4:**
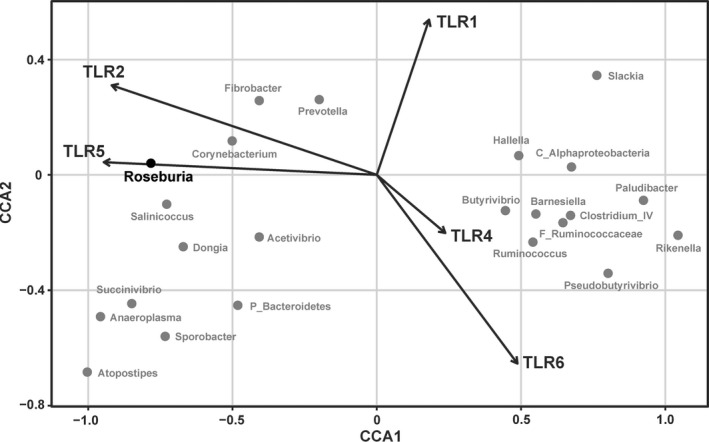
Constrained correspondence analysis revealed the correlations between the abundance of the microbial biomarkers and the expression of the significantly changed TLRs (*p *<* *.05 in *t* test)

CCA analysis (Figure 4) showed that expression of TLR5 was most highly correlated with the abundance of *Roseburia*, a major butyrate‐producing bacterium found in the human colon. There were no significant correlations between the expression of TLR2 and the abundance of other biomarkers.

## 4 DISCUSSION

In the rumen, approximately 60%–70% of the ingested feed is fermented by microbes to produce SCFAs. The major SCFAs are acetate, propionate, and butyrate. They supply approximately 50%–70% of the energy needs of the animals (Bergman, 1990). Acetate is utilized by adipose tissue (Britton & Krehbiel, 1993). Propionate is the main substrate or precursor for gluconeogenesis in the liver. The liver produces 60%–70% of the body's glucose via gluconeogenesis (Annison, Lindsay, & Nolan, 2002). Butyrate is the most preferred fuel utilized by digestive epithelial cells, providing energy for their metabolic and immune activities (Muller, Westergaard, Christensen, & Sorensen, 2002). In the ruminal microbiota, the function and the expression of the genes involved in metabolism and virulence are affected by the SCFAs concentration (Pacheco et al., 2012). Furthermore, SCFA is the principal element involved in forming the rumen osmotic pressure. Its concentration is negatively correlated with the rumen pH. Both the osmolarity and the pH directly influence the microbial composition. Therefore, the stability of the SCFAs concentrations and their molar proportions are essential for a healthy rumen ecosystem and a healthy animal. Any destruction of the homeostatic state impairs the physiology of the animal and its metabolism, as well as the survival of the ruminal microbiota. Over the course of evolution, strategies have been developed to maintain the stability of the ruminal ecosystem for both the microbiota and ruminal epithelium in the face of ongoing challenges.

There are two important indices of system stability: (1) resilience, which refers to the speed of which the system returns to its original equilibrium following interference; and (2) resistance, which refers to the ability at the ecosystem to defy functional changes when subjected to interference (Edwards et al., 2008). In a stable feeding system, individual SCFAs are produced at a stable ratio in the rumen (Sutton et al., 2003). In this study, the matutinal infusion of butyrate into the rumen induced transient changes in acetate, propionate, and butyrate proportions. Thereafter, however, the altered proportions of individual SCFAs returned to their baseline values within 5 hr. This recovery process indicated good resilience of the ruminal ecosystem. Our previous studies (Malhi et al., 2013) showed that this type of resilience of the ruminal ecosystem was mainly generated by the ruminal epithelium, which adjusted the SCFAs absorption and metabolism. We also observed that after 28 day of the butyrate infusion, the basal concentration (at 0 hr, before the butyrate infusion) of the total SCFA and the molar proportions of the individual SCFAs did not differ between groups. This phenomenon indicated good resistance of the ruminal ecosystem to interference. Thus far, the strategies used to maintain the resistance of the microbial ecosystem are far from being understood. Studies of soil microbial communities indicated that the compositions of the microbial community may exert important roles in maintenance of system resistance (Griffiths et al., 2004). In this study, butyrate interference led to significant changes in the composition of the ruminal microbiota. At the same time, a significant decrease in the ratio of Firmicutes to Bacteroidetes was observed in the microbial community. This result is consistent with the observation of the ruminal microbiota in Holstein cows that received 128 hr of continuous butyrate infusion (Li, Wu, Baldwin, Li, & Li, 2012). It has been shown in many animals that the bacteria of phyla Firmicutes and Bacteroidetes are dominant in the microbial community of the GI ecosystem, accounting for 80%–90% of the total members (Gharechahi et al., 2015). Previous studies of humans have shown that an increase in the ratio of Firmicutes to Bacteroidetes was associated with the ability to extract more butyrate as energy from food (Jumpertz et al., 2011). In this study, the exogenous butyrate induced an increase in the amount of ruminal butyrate. The decreased ratio of Firmicutes to Bacteroidetes might be a response of the microbiota to decrease the production of butyrate to maintain a stable proportion in the rumen. Additionally, LEfSe analysis revealed, after 28 day butyrate infusion, the changes of biomarker bacteria from *Butyrivibrio*,* Pseudobutyrivibrio*, and species in Clostridium IV (major butyrate‐producing bacteria in human colon, Durso et al., 2010), to *Acetivibrio* (acetate‐producing bacterium, Flint, Bayer, Rincon, Lamed, & White, 2008), *Prevotella* (propionate‐producing bacterium, Matsui et al., 2000), and *Succinivibrio* (propionate‐producing bacterium, O'Herrin & Kenealy, 1993). Such shifts might be a strategy used by the microbiota to maintain stable proportions of the major SCFAs in the rumen. Together, these data revealed that changes in the microbial composition contributed to the maintenance of the resistance of the rumen ecosystem.

It is recognized that the host immune system promotes the residence of commensal bacteria in healthy animals (Swiatczak & Cohen, 2015). In this study, the expression of TLR2, TLR5, and MyD88 was increased, but the expression of IL‐1β and IFN‐γ was decreased by the butyrate infusion. The upregulations of TLRs were simultaneously observed with the changes of the microbial composition. In healthy animals, the activation of TLRs can suppress the production of cytokines through recruiting the adaptor molecule MyD88 (Jimenez‐Dalmaroni, Gerswhin, & Adamopoulos, 2016). However, the complex suppression mechanisms are still unclear. Lukens et al. (2014) found that an increase in IL‐1β production in the gut epithelium induced the response of the intestinal resident macrophages to enteric infections in inflammation in mice. Therefore, decreasing the inappropriate immune response may increase epithelial tolerance of the nonpathogenic bacteria in healthy animals (Chu & Mazmanian, 2013). The role of the TLRs in promoting the residence of commensal bacteria in the GI ecosystem is just starting to be understood. Research has shown that TLRs on the apical surface of the epithelium (TLR1, TLR2, TLR5, TLR6, and TLR10) might be the candidates that receive the safety signals from GI microbiota. Carvalho et al. (2012) reported that TLR5 signaling promoted the expansion of nonpathogens and suppressed the bloom of pathogens in the guts of mice. Kellermayer et al. (2011) reported that TLR2 signaling affected the abundance of 22 bacteria in the guts of mice. Our study indicated that the signaling of epithelial TLR2 and TLR5 was associated with the changes of the microbial composition of the rumen. This change was caused by the exogenous butyrate interference. To the best of our knowledge, this is the first report to show that TLRs are related to the changes of the microbial composition in healthy ruminants. It also suggested that TLR signaling contributes to the stability of the GI ecosystem.

It is widely accepted that the activation of TLRs needs special external components supplied by resident microbes. However, the corresponding microbes that activated these TLRs are unknown. In our study, the abundance of *Roseburia* was increased significantly by the butyrate infusion. This increase was accompanied by a significant increase in the TLR5 expression of the rumen epithelium. CCA revealed a positive correlation between the relative abundance of *Roseburia* and the expression of TLR5, which suggested the possibility of an interaction between them. This result shows the possible interaction of the butyrate‐producing bacterium, *Roseburia*, and TLR5 in healthy animals. On the contrary, the expression of IL‐1β and IFN‐γ in the epithelium was decreased significantly after the butyrate infusion. The study of Sokol et al. (2008) showed that the butyrate‐producing bacterium *Faecalibacterium prausnitzii* suppressed the secretion of the proinflammatory cytokines in the colon of mice. Both *Faecalibacterium* and *Roseburia* are important butyrate‐producing bacteria of the human colonic ecosystem. In the GI ecosystem, butyrate synthesis by microbes can occur via butyrate kinase or via butyryl‐coenzyme A (CoA) and acetate CoA‐transferase (Louis & Flint, 2009). Interestingly, these two genera are similar in the butyrate metabolic pathway as both are characterized by butyryl‐CoA and acetate CoA‐transferase activity but not butyrate kinase activity (Duncan, Barcenilla, Stewart, Pryde, & Flint, 2002). It is possible that, in this study, the suppression of IL‐1 and IFN‐γ was caused by *Roseburia*. Thus far, the microbiota‐gardening effect of TLR5 has only been reported in TLR5 KO mice. Our results suggest that activation of the microbe‐TLR‐cytokine axis can be an important strategy used by GI commensal bacteria to maintain the resistance of the GI ecosystem.

In summary, our results indicated the existence of microbe‐TLR‐cytokine axis, which is involved in maintaining the stability of the GI ecosystem by increasing the epithelial tolerance to commensal bacteria. It enhances our knowledge of the fundamental principles of the GI ecosystem and provides new insight into the improvement of animal production and human health.

## DATABASE SUBMISSION

The sequencing data are available in the NCBI under BioProject PRJNA322589.

## CONFLICT OF INTEREST

No conflict of interest is declared by authors.

## References

[mbo3436-bib-0001] Annison, E. F. , Lindsay, D. B. , & Nolan, J. V . (2002). Digestion and metabolism. In Sheep nutrition: Chapter 5 In Freer M. & Dove H. (Eds.), Sheep nutrition (pp. 95–118). Wallingford: CABI Pub in association with CSIRO Pub.

[mbo3436-bib-0002] Bergman, E. N. (1990). Energy contributions of volatile fatty acids from the gastrointestinal tract in various species. Physiological Reviews, 70, 567–590. 2181501 10.1152/physrev.1990.70.2.567

[mbo3436-bib-0003] Britton, R. , & Krehbiel, C. (1993). Nutrient Metabolism by Gut Tissues1. Journal of Dairy Science, 76, 2125–2131. 8345135 10.3168/jds.S0022-0302(93)77547-5

[mbo3436-bib-0004] Carvalho, F. A. , Koren, O. , Goodrich, J. K. , Johansson, M. E. , Nalbantoglu, I. , Aitken, J. D. , … Gewirtz, A. T. (2012). Transient inability to manage proteobacteria promotes chronic gut inflammation in TLR5‐deficient mice. Cell Host & Microbe, 12, 139–152. 22863420 10.1016/j.chom.2012.07.004 PMC4310462

[mbo3436-bib-0005] Chu, H. , & Mazmanian, S. K. (2013). Innate immune recognition of the microbiota promotes host‐microbial symbiosis. Nature Immunology, 14, 668–675. 23778794 10.1038/ni.2635 PMC4109969

[mbo3436-bib-0006] Cragg, R. G. , & Bardgett, R. D. (2001). How changes in soil faunal diversity and composition within a trophic group influence decomposition processes. Soil Biology and Biochemistry, 33, 2073–2081.

[mbo3436-bib-0007] Duncan, S. H. , Barcenilla, A. , Stewart, C. S. , Pryde, S. E. , & Flint, H. J. (2002). Acetate utilization and butyryl coenzyme A (CoA):Acetate‐CoA transferase in butyrate‐producing bacteria from the human large intestine. Applied and Environment Microbiology, 68, 5186–5190. 10.1128/AEM.68.10.5186-5190.2002 PMC126392 12324374

[mbo3436-bib-0008] Durso, L. M. , Harhay, G. P. , Smith, T. P. , Bono, J. L. , Desantis, T. Z. , Harhay, D. M. , … Clawson, M.L . (2010). Animal‐to‐animal variation in fecal microbial diversity among beef cattle. Applied and Environment Microbiology, 76, 4858–4862. 10.1128/AEM.00207-10 PMC2901724 20472731

[mbo3436-bib-0009] Edgar, R. C. (2004). MUSCLE: A multiple sequence alignment method with reduced time and space complexity. BMC Bioinformatics, 5, 113. 15318951 10.1186/1471-2105-5-113 PMC517706

[mbo3436-bib-0010] Edwards, J. E. , Huws, S. A. , Kim, E. J. , Lee, M. R. , Kingston‐Smith, A. H. , & Scollan, N. D. (2008). Advances in microbial ecosystem concepts and their consequences for ruminant agriculture. Animal, 2, 653–660. 22443590 10.1017/S1751731108002164

[mbo3436-bib-0011] Flint, H. J. , Bayer, E. A. , Rincon, M. T. , Lamed, R. , & White, B. A. (2008). Polysaccharide utilization by gut bacteria: Potential for new insights from genomic analysis. Nature Reviews Microbiology, 6, 121–131. 18180751 10.1038/nrmicro1817

[mbo3436-bib-0101] Gharechahi, J. , Zahiri, H.S. , Noghabi, K.A. , & Salekdeh, G.H. (2015). In‐depth diversity analysis of the bacterial community resident in the camel rumen. Syst Appl Microbiol, 38, 67–76. 25467553 10.1016/j.syapm.2014.09.004

[mbo3436-bib-0012] Gressley, T. F. , Hall, M. B. , & Armentano, L. E. (2011). Ruminant nutrition symposium: Productivity, digestion, and health responses to hindgut acidosis in ruminants. Journal of Animal Science, 89, 1120–1130. 21415422 10.2527/jas.2010-3460

[mbo3436-bib-0013] Griffiths, B. S. , Kuan, H. L. , Ritz, K. , Glover, L. A. , McCaig, A. E. , & Fenwick, C. (2004). The relationship between microbial community structure and functional stability, tested experimentally in an upland pasture soil. Microbial Ecology, 47, 104–113. 15259275 10.1007/s00248-002-2043-7

[mbo3436-bib-0014] Jacobs, J. P. , & Braun, J. (2014). Immune and genetic gardening of the intestinal microbiome. FEBS Letters, 588, 4102–4111. 24613921 10.1016/j.febslet.2014.02.052 PMC4156569

[mbo3436-bib-0015] Jimenez‐Dalmaroni, M. J. , Gerswhin, M. E. , & Adamopoulos, I. E. (2016). The critical role of toll‐like receptors–From microbial recognition to autoimmunity: A comprehensive review. Autoimmunity Reviews, 15, 1–8. 26299984 10.1016/j.autrev.2015.08.009 PMC4679489

[mbo3436-bib-0016] Jumpertz, R. , Le, D. S. , Turnbaugh, P. J. , Trinidad, C. , Bogardus, C. , Gordon, J. I. , & Krakoff, J. (2011). Energy‐balance studies reveal associations between gut microbes, caloric load, and nutrient absorption in humans. American Journal of Clinical Nutrition, 94, 58–65. 21543530 10.3945/ajcn.110.010132 PMC3127503

[mbo3436-bib-0017] Kellermayer, R. , Dowd, S. E. , Harris, R. A. , Balasa, A. , Schaible, T. D. , Wolcott, R. D. , … Smith, C. W . (2011). Colonic mucosal DNA methylation, immune response, and microbiome patterns in Toll‐like receptor 2‐knockout mice. The FASEB Journal, 25, 1449–1460. 21228220 10.1096/fj.10-172205 PMC3079304

[mbo3436-bib-0018] Larsson, E. , Tremaroli, V. , Lee, Y. S. , Koren, O. , Nookaew, I. , Fricker, A. , … Backhed, F . (2012). Analysis of gut microbial regulation of host gene expression along the length of the gut and regulation of gut microbial ecology through MyD88. Gut, 61, 1124–1131. 22115825 10.1136/gutjnl-2011-301104 PMC3388726

[mbo3436-bib-0019] Li, R. W. , Wu, S. , Baldwin, R. L. T. , Li, W. , & Li, C . (2012). Interference dynamics of the rumen microbiota in response to exogenous butyrate. PLoS ONE,7, e29392. 22253719 10.1371/journal.pone.0029392 PMC3257242

[mbo3436-bib-0020] Liu, J. H. , Bian, G. R. , Zhu, W. Y. , & Mao, S. Y. (2015). High‐grain feeding causes strong shifts in ruminal epithelial bacterial community and expression of Toll‐like receptor genes in goats. Frontiers in Microbiology, 6, 167. 25784904 10.3389/fmicb.2015.00167 PMC4345813

[mbo3436-bib-0021] Livak, K. J. , & Schmittgen, T. D. (2001). Analysis of relative gene expression data using real – time quantitative PCR and the 2 ^−ΔΔ*C*T^ Method. Methods, 25, 402–408. 11846609 10.1006/meth.2001.1262

[mbo3436-bib-0022] Loreau, M. , Naeem, S. , Inchausti, P. , Bengtsson, J. , Grime, J. P. , & Hector, A ., … Wardle, D. A . (2001). Biodiversity and ecosystem functioning: Current knowledge and future challenges. Science, 294, 804–808. 11679658 10.1126/science.1064088

[mbo3436-bib-0024] Louis, P. , & Flint, H. J. (2009). Diversity, metabolism and microbial ecology of butyrate‐producing bacteria from the human large intestine. FEMS Microbiology Letters, 294, 1–8. 19222573 10.1111/j.1574-6968.2009.01514.x

[mbo3436-bib-0025] Lukens, J. R. , Gurung, P. , Vogel, P. , Johnson, G. R. , Carter, R. A. , McGoldrick, D. J. , … Kanneganti, T. D . (2014). Dietary modulation of the microbiome affects autoinflammatory disease. Nature, 516, 246–249. 25274309 10.1038/nature13788 PMC4268032

[mbo3436-bib-0102] Magoč, T. , & Salzberg, S.L. (2011). FLASH: fast length adjustment of short reads to improve genome assemblies. Bioinformatics, 27, 2957–2963. 21903629 10.1093/bioinformatics/btr507 PMC3198573

[mbo3436-bib-0026] Malhi, M. , Gui, H. , Yao, L. , Aschenbach, J. R. , Gabel, G. , & Shen, Z. (2013). Increased papillae growth and enhanced short‐chain fatty acid absorption in the rumen of goats are associated with transient increases in cyclin D1 expression after ruminal butyrate infusion. Journal of Dairy Science, 96, 7603–7616. 24119813 10.3168/jds.2013-6700

[mbo3436-bib-0027] Matsui, H. , Ogata, K. , Tajima, K. , Nakamura, M. , Nagamine, T. , Aminov, R. I. , & Benno, Y. (2000). Phenotypic characterization of polysaccharidases produced by four Prevotella type strains. Current Microbiology, 41, 45–49. 10919398 10.1007/s002840010089

[mbo3436-bib-0028] McMurdie, P. J. , & Holmes, S. (2013). phyloseq: An R package for reproducible interactive analysis and graphics of microbiome census data. PLoS ONE, 8, e61217. 23630581 10.1371/journal.pone.0061217 PMC3632530

[mbo3436-bib-0029] Mori, H. , Maruyama, F. , Kato, H. , Toyoda, A. , Dozono, A. , Ohtsubo, Y. , … Kurokawa, K . (2014). Design and experimental application of a novel non‐degenerate universal primer set that amplifies prokaryotic 16S rRNA genes with a low possibility to amplify eukaryotic rRNA genes. DNA Research, 21, 217–227. 24277737 10.1093/dnares/dst052 PMC3989492

[mbo3436-bib-0030] Muller, A. K. , Westergaard, K. , Christensen, S. , & Sorensen, S. J. (2002). The diversity and function of soil microbial communities exposed to different disturbances. Microbial Ecology, 44, 49–58. 11976785 10.1007/s00248-001-0042-8

[mbo3436-bib-0031] O'Herrin, S. M. , & Kenealy, W. R. (1993). Glucose and carbon dioxide metabolism by *Succinivibrio dextrinosolvens* . Applied and Environment Microbiology, 59, 748–755. 10.1128/aem.59.3.748-755.1993 PMC202185 8481001

[mbo3436-bib-0032] Oksanen, J. , Blanchet, F. G. , Kindt, R. , Legendre, P. , Minchin, P. R. , & O'Hara, R. B ., … Wagner, H . (2016). vegan: Community Ecology Package. R package version 2.3‐5.

[mbo3436-bib-0033] Pacheco, A. R. , Curtis, M. M. , Ritchie, J. M. , Munera, D. , Waldor, M. K. , Moreira, C. G. , & Sperandio, V. (2012). Fucose sensing regulates bacterial intestinal colonization. Nature, 492, 113–117. 23160491 10.1038/nature11623 PMC3518558

[mbo3436-bib-0034] Paradis, E. , Claude, J. , & Strimmer, K. (2004). APE: Analyses of Phylogenetics and Evolution in R language. Bioinformatics, 20, 289–290. 14734327 10.1093/bioinformatics/btg412

[mbo3436-bib-0035] Pfisterer, A. B. , & Schmid, B. (2002). Diversity‐dependent production can decrease the stability of ecosystem functioning. Nature, 416, 84–86. 11882897 10.1038/416084a

[mbo3436-bib-0036] Segata, N. , Izard, J. , Waldron, L. , Gevers, D. , Miropolsky, L. , Garrett, W. S. , & Huttenhower, C. (2011). Metagenomic biomarker discovery and explanation. Genome Biology, 12, R60. 21702898 10.1186/gb-2011-12-6-r60 PMC3218848

[mbo3436-bib-0037] Sokol, H. , Pigneur, B. , Watterlot, L. , Lakhdari, O. , Bermudez‐Humaran, L. G. , Gratadoux, J. J. , … Langella, P . (2008). Faecalibacterium prausnitzii is an anti‐inflammatory commensal bacterium identified by gut microbiota analysis of Crohn disease patients. Proceedings of the National Academy of Sciences U S A, 105, 16731–16736. 10.1073/pnas.0804812105 PMC2575488 18936492

[mbo3436-bib-0038] Stamatakis, A. (2014). RAxML version 8: A tool for phylogenetic analysis and post‐analysis of large phylogenies. Bioinformatics, 30, 1312–1313. 24451623 10.1093/bioinformatics/btu033 PMC3998144

[mbo3436-bib-0040] Sutton, J. D. , Dhanoa, M. S. , Morant, S. V. , France, J. , Napper, D. J. , & Schuller, E. (2003). Rates of production of acetate, propionate, and butyrate in the rumen of lactating dairy cows given normal and low‐roughage diets. Journal of Dairy Science, 86, 3620–3633. 14672193 10.3168/jds.S0022-0302(03)73968-X

[mbo3436-bib-0041] Swiatczak, B. , & Cohen, I. R. (2015). Gut feelings of safety: Tolerance to the microbiota mediated by innate immune receptors. Microbiology and Immunology, 59, 573–585. 26306708 10.1111/1348-0421.12318

[mbo3436-bib-0043] Wickham, H . (2009) ggplot2: Elegant Graphics for Data Analysis. New York: Springer‐Verlag.

[mbo3436-bib-0045] Yang, W. , Shen, Z. , & Martens, H. (2012). An energy‐rich diet enhances expression of Na(+)/H(+) exchanger isoform 1 and 3 messenger RNA in rumen epithelium of goat. Journal of Animal Science, 90, 307–317. 21856899 10.2527/jas.2011-3854

