# Supplementary material for: Maintaining stability of the rumen ecosystem is associated with changes of microbial composition and epithelial TLR signaling
Source: Microbiologyopen. 2017 Jan 21;6(3):e00436. doi: 10.1002/mbo3.436 (PMC5458463; doi:10.1002/mbo3.436)
Supplement: Supplementary file 1 [file MBO3-6-na-s001.docx]

Fig. S1. A) NMDS analysis of Bray-Curtis similarity coefficients based on the OTUs of each sample. B) Analysis of similarities (ANOSIM) based on the OTUs of each sample. C) Commensal diversity estimated using the Shannon and Simpson indices.





Table S1. Butyrate infusion resulted in different changes of rumen microbial composition (mean percentage ± SE; N = 3).

| Item | Genus | BT±SE | CO±SE |
| --- | --- | --- | --- |
| 1 | Prevotella | 27358±4570 | 11750±1287 |
| 2 | O_Bacteroidales | 22836±5907 | 25731±1856 |
| 3 | P_Bacteroidetes | 17338±5559 | 6345±554 |
| 4 | F_Porphyromonadaceae | 5971±2600 | 6876±891 |
| 5 | F_Prevotellaceae | 4385±818 | 8597±1846 |
| 6 | F_Ruminococcaceae | 3719±921 | 16082±2463 |
| 7 | Subdivision5_genera_incertae_sedis | 3326±1514 | 4157±1463 |
| 8 | F_Lachnospiraceae | 2267±801 | 2420±259 |
| 9 | Unclassified_Bacteria | 1118±244 | 1338±180 |
| 10 | Paraprevotella | 1082±236 | 624±74 |
| 11 | Vampirovibrio | 1061±371 | 1278±425 |
| 12 | Lachnospiracea_incertae_sedis | 1034±440 | 1491±132 |
| 13 | Victivallis | 899±257 | 900±374 |
| 14 | F_Veillonellaceae | 853±410 | 1290±650 |
| 15 | O_Clostridiales | 844±206 | 1389±122 |
| 16 | P_Firmicutes | 787±228 | 439±76 |
| 17 | Anaeroplasma | 768±214 | 26±26 |
| 18 | Fibrobacter | 603±114 | 134±22 |
| 19 | C_Lentisphaeria | 470±88 | 465±128 |
| 20 | Ruminococcus | 416±168 | 1312±222 |
| 21 | Treponema | 336±61 | 287±54 |
| 22 | Butyrivibrio | 277±140 | 859±242 |
| 23 | Barnesiella | 235±62 | 822±33 |
| 24 | Succinivibrio | 208±58 | 18±15 |
| 25 | Acetivibrio | 180±28 | 75±19 |
| 26 | TM7_genera_incertae_sedis | 165±39 | 261±63 |
| 27 | Paludibacter | 154±60 | 3004±450 |
| 28 | P_Proteobacteria | 153±76 | 116±29 |
| 29 | Oscillibacter | 138±42 | 264±91 |
| 30 | Anaerovibrio | 117±45 | 120±28 |
| 31 | Spirochaeta | 81±12 | 72±19 |
| 32 | Clostridium_IV | 76±20 | 373±40 |
| 33 | Succiniclasticum | 74±38 | 121±80 |
| 34 | Anaerovorax | 56±10 | 72±12 |
| 35 | Coprococcus | 50±27 | 4±4 |
| 36 | F_Clostridiales_Incertae_Sedis_XI | 43±15 | 26±12 |
| 37 | C_Alphaproteobacteria | 40±10 | 218±58 |
| 38 | C_Epsilonproteobacteria | 40±22 | 8±4 |
| 39 | F_Coriobacteriaceae | 38±12 | 18±12 |
| 40 | SR1_genera_incertae_sedis | 37±7 | 50±15 |
| 41 | Syntrophococcus | 36±10 | 44±15 |
| 42 | Sporobacter | 28±9 | 4±4 |
| 43 | Pseudobutyrivibrio | 25±6 | 54±4 |
| 44 | Corynebacterium | 24±2 | 0±0 |
| 45 | Roseburia | 20±8 | 0±0 |
| 46 | Hallella | 15±2 | 50±16 |
| 47 | Dongia | 14±3 | 0±0 |
| 48 | Salinicoccus | 12±2 | 2±2 |
| 49 | Atopostipes | 12±5 | 0±0 |
| 50 | Atopobium | 11±6 | 2±2 |
| 51 | Desulfovibrio | 11±7 | 0±0 |
| 52 | C_Deltaproteobacteria | 10±10 | 22±5 |
| 53 | Saccharofermentans | 10±6 | 18±10 |
| 54 | Selenomonas | 9±5 | 2±2 |
| 55 | Oribacterium | 8±4 | 14±2 |
| 56 | Blautia | 8±8 | 10±7 |
| 57 | Eubacterium | 8±8 | 0±0 |
| 58 | Olsenella | 7±7 | 0±0 |
| 59 | Acetanaerobacterium | 6±3 | 6±3 |
| 60 | F_Neisseriaceae | 6±3 | 6±3 |
| 61 | Guggenheimella | 6±6 | 0±0 |
| 62 | Alkalibacterium | 6±3 | 0±0 |
| 63 | F_Erysipelotrichaceae | 5±5 | 47±35 |
| 64 | Schwartzia | 5±5 | 2±2 |
| 65 | O_Actinomycetales | 5±3 | 2±2 |
| 66 | O_Bacillales | 5±3 | 2±2 |
| 67 | Clostridium_XlVb | 4±4 | 2±2 |
| 68 | Flavonifractor | 4±4 | 2±2 |
| 69 | F_Desulfovibrionaceae | 4±4 | 2±2 |
| 70 | Pseudoflavonifractor | 4±4 | 2±2 |
| 71 | Pseudomonas | 4±4 | 0±0 |
| 72 | Slackia | 3±3 | 48±24 |
| 73 | Cerasicoccus | 3±3 | 6±3 |
| 74 | Lysinibacillus | 3±3 | 6±3 |
| 75 | Yaniella | 3±3 | 0±0 |
| 76 | Neisseria | 3±3 | 0±0 |
| 77 | Rikenella | 2±2 | 58±26 |
| 78 | F_Synergistaceae | 2±2 | 43±23 |
| 79 | F_Sutterellaceae | 2±2 | 4±4 |
| 80 | Desulfobulbus | 2±2 | 2±2 |
| 81 | Elusimicrobium | 2±2 | 0±0 |
| 82 | Moraxella | 2±2 | 0±0 |
| 83 | F_Thermoactinomycetaceae | 2±2 | 0±0 |
| 84 | Enterorhabdus | 2±2 | 0±0 |
| 85 | Rhodanobacter | 2±2 | 0±0 |
| 86 | Bilophila | 2±2 | 0±0 |
| 87 | F_Anaerolineaceae | 0±0 | 24±14 |
| 88 | Acholeplasma | 0±0 | 8±4 |
| 89 | Mogibacterium | 0±0 | 6±3 |
| 90 | F_Enterobacteriaceae | 0±0 | 4±4 |
| 91 | O_Clostridiales | 0±0 | 4±4 |
| 92 | Gemmatimonas | 0±0 | 4±4 |
| 93 | Streptococcus | 0±0 | 4±4 |
| 94 | Moryella | 0±0 | 4±4 |
| 95 | F_Flavobacteriaceae | 0±0 | 4±4 |
| 96 | Armatimonadetes_gp1 | 0±0 | 4±4 |
| 97 | Elioraea | 0±0 | 4±4 |
| 98 | O_Myxococcales | 0±0 | 4±4 |
| 99 | Ignavibacterium | 0±0 | 4±4 |
| 100 | Alkaliphilus | 0±0 | 2±2 |
| 101 | Bacteroides | 0±0 | 2±2 |
| 102 | Escherichia/Shigella | 0±0 | 2±2 |
| 103 | F_Microbacteriaceae | 0±0 | 2±2 |
| 104 | Curtobacterium | 0±0 | 2±2 |
| 105 | F_Succinivibrionaceae | 0±0 | 2±2 |
| 106 | Luteibacter | 0±0 | 2±2 |
| 107 | Streptomyces | 0±0 | 2±2 |

The consensus sequence of each Genus was annotated to the closest lineage blasting against RDA database.

F: = family; O: = order; C: = class; P: = phylum.

Genera were sorted based on their relative abundance in the rumen microbial community in a descending order.

Table S2. Chemical Compositions of Diet

| Chemical composition | Concentrate | Hay |
| --- | --- | --- |
| DM (%) | 87.75 | 89.81 |
| Crude protein (% of DM) | 20.89 | 7.32 |
| Crude fat (% of DM) | 3.64 | 2.02 |
| Crude fiber (% of DM) | 6.67 | 28.25 |
| Crude ash (% of DM) | 7.73 | 6.4 |
| ME (MJ/kg of DM) | 10.85 | 6.96 |

The concentrate was composed of ground corn, soybean meal, cottonseed bran, wheat bran, fish meal, calcium phosphate, limestone, trace mineral salt, and vitamin premix (vitamins A, D, and E).

### Table S3 Quantitative PCR primers used in this study

| Genes | Genbank Nr. | Forward (5'>3') | Reverse (5'>3') |
| --- | --- | --- | --- |
| GAPDH | HM043737.1 | TTGTCTCCTGCGACTTCA | CCACCACCCTGTTACTGTT |
| TLR1 | NM_001285605.1 | ACAATCCATTCCAATGTTCC | ACAATGGTGACAATCAGCAG |
| TLR2 | XM_013970466.1 | CTGTGTGCGTCTTCCTCAGA | TCAGGGAGCAGAGTAACCAGA |
| TLR4 | NM_001285574.1 | GGTTTCCACAAAAGCCGTAA | AGGACGATGAAGATGATGCC |
| TLR5 | NM_001285699.1 | ACCTGGGTGGAAGTCAGATA | GGTTGAGGGAAAAATCAATG |
| TLR6 | NM_001285540.1 | AGGCCAAGTATCAAGAGACG | AGAGGACAGTCACAGCAACA |
| TLR10 | NM_001285541.1 | TTGCATGATGGAATCAAAAC | AACCAATTGGAAGATGAGGA |
| MyD88 | XM_013973392.1 | ACAAGCCAATGAAGAAAGAG | GAGGCGAGTCCAGAACC |
| IFN-γ | NM_001285682.1 | TGATTCAAATTCCGGTGGAT | GCAGGCAGGAGAACCATTAC |
| IL-1ß | 窗体顶端  XM_013967700.1窗体底端 | CATGTGTGCTGAAGGCTCTC | AGTGTCGGCGTATCACCTTT |
| IL-6 | NM_001285640.1 | CCAATCTGGGTTCAATCAGG | ACCCACTCGTTTGAGGACTG |
| IL-10 | XM_005690416.2 | TTAAGGGTTACCTGGGTTGC | CCCTCTCTTGGAGCATATTGA |
| TNF-α | NM_001286442.1 | CAAGTAACAAGCCGGTAGCC | AGATGAGGTAAAGCCCGTCA |

### GAPDH = glyceraldehyde 3 phosphate dehydrogenase; TLR = toll like receptor; MyD88 = myeloid differentiation primary response 88; IFN-γ = interferon- gamma; IL = iterleukin; TNF-α = tumor necrosis factor alpha

The reference sequence number is given for primers whose source is the National Center for Biotechnology Information (NCBI) GenBank database (http://www.ncbi.nlm.nih.gov/genbank/).
